# Non-Disclosure of HIV Status and Associations with Psychological Factors, ART Non-Adherence, and Viral Load Non-Suppression Among People Living with HIV in the UK

**DOI:** 10.1007/s10461-016-1541-4

**Published:** 2016-09-01

**Authors:** Marina Daskalopoulou, Fiona C. Lampe, Lorraine Sherr, Andrew N. Phillips, Margaret A. Johnson, Richard Gilson, Nicky Perry, Ed Wilkins, Monica Lascar, Simon Collins, Graham Hart, Andrew Speakman, Alison J. Rodger, For the ASTRA Study Group

**Affiliations:** 1 Department of Infection and Population Health, University College London Royal Free Hospital, London, UK; 2 Department of Primary Care and Population Health, University College London, London, UK; 3 Royal Free Centre for HIV Medicine, Ian Charleson Day Centre, Royal Free Hospital, London, UK; 4 Brighton and Sussex University Hospitals NHS Trust, Brighton, UK; 5 Pennine Acute Hospitals NHS Trust, Manchester, UK; 6 Barts Health NHS Trust, London, UK; 7 HIV iBase, London, UK

**Keywords:** HIV, Antiretroviral, Adherence, Disclosure, Social support

## Abstract

Disclosure of HIV status to family, friends, and a stable partner may be linked to improved health outcomes for people living with HIV. This study assessed whether non-disclosure is associated with psychological symptoms, non-adherence to antiretroviral therapy (ART), and viral load (VL) non-suppression. A total of 3258 HIV-diagnosed individuals in the UK completed the confidential ASTRA study questionnaire (2011–2012). Participants reported whether they told anyone they had HIV; to which confidant(s) (friends, family, work colleagues, stable partner) and to what extent (none, some, most/all). The prevalence and factors associated with non-disclosure were assessed. Associations between non-disclosure and the following factors were established using modified Poisson regression with adjustment for socio-demographic factors (gender, age group, ethnicity), HIV-related factors (time since HIV diagnosis, ART status), and clinic: low social support (score ≤ 12 on modified Duke-UNC FSSQ); depression and anxiety symptoms (≥10 on PHQ-9 and GAD-7 respectively); self-reported ART non-adherence in past 2 weeks/3 months; VL non-suppression (clinic-recorded VL > 50 copies/mL among those who started ART ≥ 6 months ago). Among 3233 participants with disclosure data, the prevalence of non-disclosure to anyone was 16.6 % (n/N = 61/367) among heterosexual men, 15.7 % (98/626) among women, and 5.0 % (113/2240) among MSM. MSM were more likely to disclose to some/all friends compared to family (85.8 vs. 59.9 %) while heterosexuals were less likely to disclose to friends than family (44.1 vs. 61.1 % for men, 57.5 vs. 67.1 % for women). Among 1,631 participants with a stable partner, non-disclosure to a stable partner was 4.9 % for MSM, 10.9 % for heterosexual men, and 13.0 % for women. In adjusted analyses, older age (≥60 years), non-white ethnicity, more recent HIV diagnosis, and not having a stable partner were significantly associated with overall non-disclosure for MSM and heterosexual individuals. The prevalence of low social support was 14.4 %, of depression and anxiety symptoms 27.1 and 22.0 %, respectively, of ART non-adherence 31.8 %, and of viral load non-suppression on ART 9.8 %. There was no evidence that non-disclosure overall (versus disclosure to anyone) was associated with low social support, depression or anxiety symptoms, ART non-adherence or VL non-suppression among MSM or heterosexual individuals. However, compared to MSM who disclosed to ‘none’ or ‘some’ friends and family, MSM who disclosed to ‘most or all’ of their friends and family were more likely to have symptoms of depression (adjusted PR = 1.4, 95 % CI 1.2–1.7), anxiety (1.3, 1.1–1.6), and to report ART non-adherence (1.3, 1.1–1.5). In this large multicentre study of people living with HIV in the UK, non-disclosure was overall low, but higher for heterosexual individuals compared to MSM. Non-disclosure was not associated with higher prevalence of adverse health measures.

**Electronic supplementary material:**

The online version of this article (doi:10.1007/s10461-016-1541-4) contains supplementary material, which is available to authorized users.

## Introduction

Disclosure of HIV status to family and friends is a selective and often planned behaviour, which balances risks and benefits [1]. Important deterrents of disclosure include the lack of a social network [2], fears of stigma and abuse [2–4], subsequent disclosure of stigmatised behaviours (sexual orientation, injecting drug use), fear of conflicts with a partner [5, 6], loss of social support, breach of confidentiality [2], and the need to not burden family members with one’s health issues [2, 7]. Additionally, HIV positive people may need time to come to terms with their own diagnosis or may feel their health is a private matter [7, 8]. Although these responses are common, disclosure may also provide a way of obtaining social and psychological support to overcome a diagnosis that is in many ways more complex than other chronic conditions [9].

Over the last decade, UK studies among HIV-outpatient clinic attendees showed that the prevalence of non-disclosure is lower among MSM compared to heterosexual individuals [2, 10–12]. Economic and educational disadvantage along with belonging to a racial/ethnic minority were associated with higher prevalence of non-disclosure in UK and US studies [1, 2, 9, 11–14]. Compared to MSM, black African heterosexuals in the UK have been shown to have higher non-disclosure rates overall, and particularly for non-disclosure to their stable partner [6, 11, 14]. Non-disclosure to a stable partner differs from non-disclosure in the social context as such a partnership usually involves a sexual relationship and concerns about the potential risk of HIV transmission. Additionally, disclosure within a relationship could be associated with a closer and more trusting relationship and improved social support [2].

Although studies show a significant minority of people with HIV choose to not disclose their status to family and friends and/or partners, the extent to which such non-disclosure impacts on psychological health, ability to adhere to antiretroviral treatments (ART), and virological outcome of ART is unclear. Associations of non-disclosure with depression or anxiety may be mediated by low social support [3]. While HIV status disclosure has been seen as an important step towards enhancing mental health through increased social support [15], evidence remains mixed [11, 12, 14, 16].

Evidence for a link between non-disclosure and non-adherence to ART is also limited. The relationship is likely to be dependent on specific socio-demographic groups surveyed, availability of ART, health care-related factors, and level of engagement in HIV care [17–21]. Thus, the implications of non-disclosure of HIV status with regards to ART adherence and virological outcomes may be different in the current era of simpler treatments and excellent prognosis for HIV.

This paper presents data from a large multicentre study of HIV-diagnosed people in the UK, which aimed to (1) determine the prevalence of non-disclosure within the social network (family, friends, co-workers) and to a current stable partner, (2) establish associations of socio-demographic and HIV-related factors with prevalence of non-disclosure in the social network and to a stable partner, and (3) examine associations of non-disclosure with: low social support, psychological symptoms, non-adherence to ART, and viral load non-suppression among those on ART.

## Methods

### ASTRA Study

The Antiretrovirals Sexual Transmission Risks and Attitudes (ASTRA) study recruited men and women with diagnosed HIV infection attending one of eight UK HIV outpatient clinics during 2011–2012 [22]. Participants completed a confidential, gender-specific questionnaire that included socio-demographic information (gender, sexual orientation, age, ethnicity, education, employment, religion, stable partner, social support), HIV-related factors (date of HIV diagnosis, ART use, ART adherence), mental health symptoms (anxiety, depression). Clinic-recorded viral load (VL) and CD4 count were documented for all participants (the latest value communicated to the participant). Low functional social support (lack of a supportive social network) was defined as a total score of 12 or less on the modified Duke-UNC FSSQ [23]. (ebox1 Supplementary Material) Depression and anxiety symptoms were defined as scores of 10 or higher on the PHQ-9 and GAD-7, respectively [24, 25]. ART non-adherence was defined (among those on ART) as missing one or more doses of ART in the previous two weeks or more than two consecutive days of ART on one or more occasions in the previous 3 months. Viral load non-suppression was defined as clinic-recorded VL > 50 copies/mL among those who had started ART at least 6 months prior to the VL measure.

Participants were asked if they were currently in an on-going relationship with a partner, defined as a wife/husband, civil partner, or girlfriend/boyfriend. They also provided information on the current partner’s HIV status, length of time in the relationship, and cohabitation.

### Disclosure in a Social Context

Participants were asked whether they had told anyone apart from health care staff that they have HIV. If they answered “Yes”, they specified whether they had told family members, friends and, if applicable, work colleagues, or a stable partner (see below disclosure to stable partner/spouse). If participants had not disclosed to anyone in these categories they were classified as not having disclosed to anyone in a social context.

### Disclosure to Family/Friends

Among those who indicated having disclosed to at least one person, participants specified whether they told “none”, “some”, or “most or all” family members or friends. A combined variable (‘disclosure to friends and family’) was defined with three categories; “none”, if participants had not disclosed to any friends or family, “most or all” if participants disclosed to “most or all” of their friends and “most or all” of their family members and “some” otherwise (for full details see Table 1).

**Table 1:** Prevalence of HIV status disclosure by gender/sexual orientation and confidant (N = 3,233)

	MSM (N = 2240)		Heterosexual men (N = 367)		Women (N = 626)	
	n	%	n	%	n	%
Overall disclosure status (N = 3233)						
No one	113	5.0	61	16.6	98	15.7
At least one person	2127	95.0	306	83.4	528	84.3
	p < 0.001					
Family members (N = 2645)						
None	754	40.1	105	38.9	162	32.9
Some	613	32.6	118	43.7	233	47.3
Most or all	515	27.4	47	17.4	98	19.9
	p < 0.001					
Friends (N = 2641)						
None	283	14.2	133	55.9	173	42.5
Some	1110	55.6	90	37.8	200	49.1
Most or all	603	30.2	15	6.3	34	8.4
	p < 0.001					
Family and friends combined (N = 2845)^a^						
None	261	12.9	100	34.8	149	28.3
Some	1401	69.0	175	61.0	349	66.2
Most or all	369	18.2	12	4.2	29	5.5
	p < 0.001					
Colleagues (N = 1400 employed)						
None	621	54.2	79	84.0	135	84.4
Some	452	39.4	13	13.8	21	13.1
Most or all	73	6.4	2	2.1	4	2.5
	p < 0.001					
Stable partner/spouse (N = 1631 in a relationship)						
Not disclosed	54	4.9	25	10.9	38	13.0
Disclosed	1056	95.1	204	89.1	254	87.0
	p < 0.001					

Information on non-disclosure overall was available for 3233 out of 3258 ASTRA participants

^a^Combination categories created from disclosure to friends and disclosure to family variables as follows; none: none in both, or no answer in one and none in the other; some: some in at least one of the two variables, or most/all in one and either no answer, none, or some in the other; most/all: both variables most/all. Missing for 217 MSM, 86 heterosexual men, 110 women

P values by Chi squared test

### Disclosure to Stable Partner/Spouse

Non-disclosure to a stable partner was defined as a “No” to the question “I have told a partner/wife/husband that I have HIV” and was assessed only among those who indicated being in an ongoing relationship with a partner.

### Disclosure in the Workplace

Among participants reporting current full- or part-time employment, non-disclosure in the workplace was defined as having disclosed to “none” of their work colleagues.

The variables disclosure to; family/friends, a stable partner, and the workplace, have a higher proportion of missing values compared to the overall disclosure variable, as a number of participants who indicated disclosure to at least one person did not provide information on type of confidant, and we did not assume that a missing answer indicated non-disclosure.

### Statistical Analysis

Prevalence of disclosure was assessed overall (no one, at least one person), by confidant (family, friends, co-workers, stable partner), and extent (none, some, most or all) among MSM, heterosexual men, and heterosexual women. Prevalence was compared across gender/sexual orientation groups using chi-squared tests.

We examined the association of socio-demographic and HIV-related factors with non-disclosure in the social context (versus disclosure to at least one person). As initial analyses suggested similar patterns of associations for heterosexual men and women, these were combined and analyses were conducted separately for MSM and heterosexual individuals. Modified Poisson regression models with cluster-robust error variances were used to produce unadjusted and adjusted prevalence ratios (PRs) with 95 % confidence intervals [26]. The association of each socio-demographic and HIV-related factor was assessed in a separate multivariable model with adjustment for gender (for the heterosexual analysis), age group (<30, 30–39, 40–49, 50–59, ≥60), ethnicity (white, black African, all other/missing), time since HIV diagnosis (≤3 months, 3 months–2 years, 2–5, 5–15, and >15 years), ART status (on ART, not on ART), and clinic.

Modified Poisson regression was used to examine the unadjusted and adjusted association of socio-demographic, HIV-related, and relationship-related factors [stable partner’s HIV status (HIV-positive, HIV-negative, unknown HIV-serostatus), time in relationship (≤2, 3–5, ≥6 years), cohabitation with partner (yes, no)] with non-disclosure to a partner/spouse (versus disclosure to partner), among participants in a stable relationship. Multivariable models included gender (for heterosexuals analysis only), age (<50, ≥50 years), ethnicity (white, all other/missing), time since HIV diagnosis (≤2, 3–10, >10 years), ART status (on or not on ART), and clinic as detailed above.

We examined the associations of two disclosure variables (overall, to friends and family) with: (1) low social support, (2) symptoms of depression, (3) symptoms of anxiety (4) non-adherence to ART, and (5) viral load (VL) non-suppression among participants on ART for at least 6 months prior to the questionnaire VL measure. Modified Poisson regression was used to examine adjusted associations; in each case the model included, in addition to the disclosure variable, age group (<50, ≥50, missing), gender (for heterosexual models only), ethnicity (white, all other/missing), time since HIV diagnosis (<2, 2–10, >10 years), and ART status (on ART, off ART; for social support, depression, anxiety analyses only), and clinic. Analyses were performed using Stata version 13.0.

## Results

### Study Population

During the study period 5112 HIV-diagnosed men and women were invited to participate in ASTRA, of whom 3258 completed the questionnaire (response rate 64 %) [22.] The mean age was 45.2 years (SD 9.6). Men who have sex with men accounted for 69.0 % (n = 2248) of respondents, of whom 89.3 % identified as white. Among heterosexual men (n = 354), 57.6 % identified as black African/other, as did 73.6 % of women (total n = 606). Overall, 44.6 % of MSM and 35.5 % of heterosexual individuals had a university degree or higher. The median time since HIV diagnosis was 9 years (IQR 4-15). Eighty-five percent of participants were currently on ART, of whom 86.6 % had suppressed viral load (≤50 copies/mL).

### Disclosure—Social Context

Information on HIV status disclosure was provided by 3233 (99.2 % of 3258 ASTRA) participants. Of these, 8.4 % (n = 272) had not disclosed their status to anyone. There were significant differences in disclosure between gender/sexual orientation groups. (Table 1) Prevalence of non-disclosure was higher in heterosexual men and women compared to MSM (16.6, 15.7, 5.0 %, respectively, p < 0.001). For MSM the prevalence of non-disclosure to family members was higher than the prevalence of non-disclosure to friends (40.1 vs. 14.2 %). The opposite pattern was observed for heterosexuals (non-disclosure to family 35.0 vs. to friends 47.4 %). A total of 1806 participants were currently employed; among the 1,400 who provided information on workplace disclosure, 84.0 % of heterosexuals and 54.2 % of MSM reported non-disclosure to any work colleagues.

A total of 1810 (56.1 %) participants had a stable partner or spouse, of whom 1631 (90.1 %) provided information on disclosure to partner (Table 1). Prevalence of non-disclosure to a stable partner was much lower among MSM (4.9 %) than among heterosexual men (10.9 %) and women (13.0 %) (p < 0.001).

### Factors Associated with Non-Disclosure in the Social Context

In unadjusted associations among 2240 MSM, using modified Poisson regression models, non-disclosure in the social context was associated with black African or other non-white ethnicity, being religious, non UK-birth, more recent HIV diagnosis (with a trend of lower non-disclosure with longer time since diagnosis), not being on ART, and not having a stable partner (p < 0.05 for all, Table 2). Although there was no significant trend with age in unadjusted analysis, prevalence of non-disclosure tended to be higher among those aged ≥60 years, compared to all other age groups. Education and employment status were not associated with non-disclosure (p > 0.05). In multivariable models (adjusted for age group, ethnicity, time since HIV diagnosis, ART status, and clinic), older age, black African and other non-white ethnicity, more recent HIV diagnosis, and not having a stable partner were independently associated with non-disclosure (p < 0.05) (Table 2).

**Table 2:** Association of socio-demographic and HIV-related factors with non-disclosure in the social context (n = 3233)

	MSM (n = 2240)						Heterosexual men and women (n = 993)					
	n not disclosed/N	%	Unadjusted PR [95 % CI]	*p* value^◊^	Adjusted PR [95 % CI]	p-value^◊^	n not disclosed/N	%	Unadjusted PR [95 % CI]	p-value^◊^	Adjusted PR [95 % CI]	p-value^◊^
Gender (N = 3233)												
Woman			–				61/367	16.6	1.0		1.0	
Man							98/626	15.7	0.9 [0.7, 1.3]	0.688	1.0 [0.7, 1.4]	0.914
Age at recruitment, years (N = 3160)												
≤30	7/109	6.4	1.0		1.0		7/62	11.3	1.0		1.0	
30–39	19/503	3.8	0.6 [0.2, 1.3]		0.9 [0.4, 2.1]		38/236	16.1	1.4 [0.7, 3.0]		1.4 [0.6, 3.0]	
40–49	47/943	5.0	0.8 [0.4, 1.7]		1.6 [0.7, 3.6]		63/412	15.3	1.4 [0.6, 2.8]		1.6 [0.7, 3.3]	
50–59	21/509	4.1	0.9 [0.4, 1.9]		1.7 [0.7, 4.0]		30/173	17.3	1.5 [0.7, 3.3]		1.7 [0.8, 3.8]	
≥60	16/153	10.5	1.6 [0.7, 3.8]	0.162(t)	4.9 [2.0, 11.9]	0.001(t)	12/60	20.0	1.8 [0.7, 4.2]	0.246(t)	2.2 [0.9, 5.3]	0.045(t)
Ethnicity (N = 3233)												
White	78/1969	4.0	1.0		1.0		25/242	10.3	1.0		1.0	
Black African	4/20	20.0	5.0 [2.0, 12.5]		4.0 [1.6, 9.9]		97/583	16.6	1.6 [1.1, 2.4]		1.6 [1.1, 2.5]	
All other (incl. missing)	31/251	12.4	3.1 [2.1, 4.6]	<0.001	3.0 [2.0, 4.6]	<0.001	37/168	22.0	2.1 [1.3, 3.4]	0.006	2.0 [1.2, 3.3]	0.015
Religious^a^ (N = 3187)												
Yes	46/1263	3.6	1.8 [1.2, 2.6]		1.4 [0.9, 2.0]		23/147	15.7	1.0 [0.7, 1.5]		0.7 [0.5, 1.2]	
No	61/940	6.5	1.0	0.002	1.0	0.103	135/837	16.1	1.0	0.883	1.0	0.193
Place of birth (N = 3191)												
UK	66/1537	4.3	1.0		1.0		25/214	11.7	1.0		1.0	
Non-UK	47/703	6.7	1.6 [1.1, 2.2]	0.017	1.2 [0.8, 1.8]	0.429	124/737	16.8	1.4 [1.0, 2.2]	0.075	1.4 [0.8, 2.3]	0.230
Education (N = 3191)												
No qualifications/up to A levels	62/1219	5.1	1.0		1.0		107/664	16.1	1.0		1.0	
University degree or higher	44/979	4.5	0.9 [0.6, 1.3]	0.520	1.1 [0.8, 1.6]	0.541	52/329	15.8	1.0 [0.7, 1.3]	0.901	1.0 [0.8, 1.4]	0.832
Employment (N = 3140)												
Employed full- or part-time	64/1355	4.7	1.0		1.0		77/452	17.0	1.0		1.0	
Unemployed	13/317	4.1	0.9 [0.5, 1.6]		0.8 [0.4, 1.4]		39/257	15.2	0.9 [0.6, 1.3]		0.9 [0.6, 1.3]	
Other (carer, retired, student)	27/518	5.2	1.1 [0.7, 1.7]	0.764	1.1 [0.7, 1.7]	0.605	30/241	12.5	0.7 [0.5, 1.1]	0.288	0.8 [0.5, 1.3]	0.623
Time since HIV diagnosis (N = 3207)												
≤3 months	9/59	15.3	7.0 [3.2, 15.5]		6.5 [2.7, 15.5]		7/20	35.0	2.8 [1.4, 5.7]		2.9 [1.2, 6.8]	
3 months–2 years	14/184	7.6	3.5 [1.7, 7.2]		4.2 [2.0, 9.2]		26/109	23.9	1.9 [1.1, 3.2]		2.1 [1.2, 3.7]	
2–5 years	25/337	7.4	3.4 [1.8, 6.5]		4.0 [2.0, 7.9]		29/163	17.8	1.4 [0.8, 2.3]		1.6 [0.9, 2.9]	
5–15 years	50/1006	5.0	2.3 [1.3, 4.1]		2.7 [1.5, 4.9]		72/512	14.1	1.1 [0.7, 1.7]		1.2 [0.7, 2.0]	
>15 years	14/643	2.2	1.0	<0.001(t)	1.0	<0.001(t)	22/174	12.6	1.0	0.001(t)	1.0	0.001(t)
ART status (N = 3199)												
Not on ART	26/332	7.8	1.7 [1.1, 2.6]		1.2 [0.7, 2.0]		21/100	21.0	1.4 [0.9, 2.1]		1.1 [0.7, 1.8]	
On ART	87/1900	4.6	1.0	0.013	1.0	0.526	133/867	15.3	1.0	0.135	1.0	0.635
Stable partner (N = 3207)												
No	73/1011	7.2	2.4 [1.6, 3.5]		2.1 [1.4, 3.1]		103/391	26.3	2.9 [2.1, 3.9]		2.8 [2.1, 3.9]	
Yes	37/1209	3.1	1.0	<0.001	1.0	<0.001	54/596	9.1	1.0	<0.001	1.0	<0.001

*PR* prevalence ratio, *CI* confidence interval

^a^Identifies as belonging to a religion (Islam, Christianity, Judaism, Hinduism, Buddhism, Sikhism, other); Adjusted PRs by separate modified Poisson regression models with adjustment for age group, ethnicity, time since HIV diagnosis, ART status, clinic, (and gender in heterosexual analysis only)

^◊^ Global p-values by Wald test; (t): test for trend

In unadjusted analyses among 993 heterosexual men and women, black or other non-white ethnicity, more recent HIV diagnosis, and not having a stable partner were associated with non-disclosure (p < 0.05 for all, Table 2); the association with non-UK birth was of borderline statistical significance. Gender, age group, being religious, education, employment, and ART status were not associated with non-disclosure. In multivariable modified Poisson models (adjusted for gender, age group, ethnicity, time since HIV diagnosis, ART status, and clinic), older age, black African and all other non-white ethnicity, more recent HIV diagnosis, and not having a stable partner remained significantly associated with non-disclosure among heterosexual individuals. (p < 0.05 for all).

### Non-Disclosure to a Stable Partner/Spouse

Disclosure status to a stable partner was available for 1110 MSM in a stable relationship. In unadjusted analysis, non-white ethnicity, more recent HIV diagnosis, not being on ART, and having a stable partner of unknown HIV status were significantly associated with non-disclosure to a stable partner (p < 0.05 for all, Table 3). After adjustment for age, ethnicity, time since HIV diagnosis, ART status, and clinic, non-white ethnicity and having a stable partner of unknown HIV-serostatus remained significantly associated with non-disclosure to a stable partner (p < 0.05 for both). Associations with more recent HIV diagnosis and not being on ART were of borderline significance. Length of time in the current relationship and cohabitation with the stable partner were not associated with partner non-disclosure among MSM.

**Table 3:** Association of socio-demographic and HIV-related factors with non-disclosure to a stable partner (n = 1637)

	MSM (n = 1110)						Heterosexual men and women (n = 527)					
	n not disclosed/N	%	Unadjusted PR [95 % CI]	p-value^◊^	Adjusted PR‡ [95 % CI]	p-value^◊^	n not disclosed/N	%	Unadjusted PR [95 % CI]	p-value^◊^	Adjusted PR [95 % CI]	p-value^◊^
Gender (N = 1637)												
Woman	–				–		38/292	13.0	1.0		1.0	
Man							25/229	10.9	0.9 [0.6, 1.3]	0.468	1.2 [0.7, 2.0]	0.491
Age, years (N = 1604)												
<50	38/776	4.9	1.0		1.0		51/392	13.0	1.0		1.0	
≥50	15/326	4.6	1.0 [0.6, 1.8]	0.998 (t)	1.2 [0.6, 2.2]	0.578 (t)	12/109	11.0	1.1 [0.4, 3.3]	0.744(t)	1.4 [0.5, 4.4]	0.547(t)
Ethnicity (N = 1627)												
White	40/983	4.1	1.0		1.0		12/142	8.5	1.0		1.0	
All other (black, Asian, mixed, other, missing)	11/116	9.5	2.3 [1.2, 4.3]	0.011	2.3 [1.2, 4.3]	0.012	53/385	13.8	1.6 [0.9, 2.9]	0.136	1.4 [0.7, 2.8]	0.282
Years since HIV diagnosis (N = 1624)												
≤2	10/112	8.9	2.5 [1.2, 5.1]		2.0 [0.9, 4.5]		10/67	14.9	1.5 [0.7, 3.2]		1.4 [0.6, 3.2]	
3–10	25/450	5.6	1.5 [0.8, 2.6]		1.4 [0.7, 2.6]		36/276	13.0	1.4 [0.8, 2.4]		1.1 [0.6, 2.0]	
>10	17/540	3.1	1.0	0.020 (t)	1.0	0.090 (t)	18/177	10.2	1.0	0.184(t)	1.0	0.494t)
ART status (N = 1,622)												
On ART	14/157	8.9	1.0		1.0		9/52	17.3	1.0		1.0	
Not on ART	40/949	4.2	2.1 [1.2, 3.8]	0.012	1.7 [0.9, 3.4]	0.099	53/464	11.4	1.4 [0.7, 2.8]	0.353	1.2 [0.5, 2.6]	0.655
Stable partner’s HIV serostatus (N = 1596)												
HIV-positive	17/468	3.6	1.0		1.0		27/208	13.0	1.0		1.0	
HIV-negative	28/581	4.8	1.3 [0.7, 2.4]		1.2 [0.6, 2.1]		21/266	7.9	0.6 [0.4, 1.1]		0.6 [0.3, 1.0]	
Unknown HIV status	6/37	16.2	4.5 [1.9, 10.6]	0.003	5.1 [2.1, 12.2]	<0.001	13/36	36.1	2.6 [1.4, 4.6]	<0.001	2.4 [1.3, 4.4]	<0.001
Years in current stable relationship (N = 1576)												
≤2	10/213	4.7	1.0		1.0		18/91	19.8	1.0		1.0	
3–5	13/226	5.8	1.2 [0.5, 2.7]		1.2 [0.5, 2.9]		11/105	10.5	0.6 [0.3, 1.1]		0.5 [0.2,1.0]	
≥6	26/637	4.1	0.9 [0.4, 1.8]	0.530 (t)	1.3 [0.6, 2.8]	0.498 (t)	31/304	10.2	0.5 [0.3, 0.8]	0.016(t)	0.5 [0.3, 0.9]	0.063(t)
Cohabitation with stable partner (N = 1630)												
No	12/276	4.3	1.0		1.0		31/179	17.3	1.0		1.0	
Yes	41/833	4.9	1.5 [0.8, 2.9]	0.208	1.2 [0.7, 2.3]	0.526	32/342	9.4	0.6 [0.3, 0.9]	0.010	0.6 [0.4, 0.9]	0.028

*PR* prevalence ratio, *CI* confidence interval

^◊^ global p-values by Wald test; (t) test for trend; adjusted PRs by separate modified Poisson regression models with adjustment for age group, ethnicity, time since HIV diagnosis, ART status, clinic, (and gender in heterosexual analysis only)

For 527 heterosexual men and women in a stable relationship, non-disclosure to a partner was associated with having a stable partner of unknown HIV status, shorter duration of relationship, and not cohabitating with the stable partner in unadjusted analysis.(p < 0.05 for all, Table 3) These associations were similar or slightly attenuated in the multivariable models (adjusted for gender, age group, ethnicity, time since HIV diagnosis, ART status, and clinic); having a stable partner of unknown HIV status and cohabitation remained significantly associated with non-disclosure, and shorter duration of relationship was of borderline significance.

### Levels of Non-Disclosure and Association with Social Support, Psychological Symptoms, ART Non-Adherence, and Virological Non-Suppression on ART

**Fig. 1:**
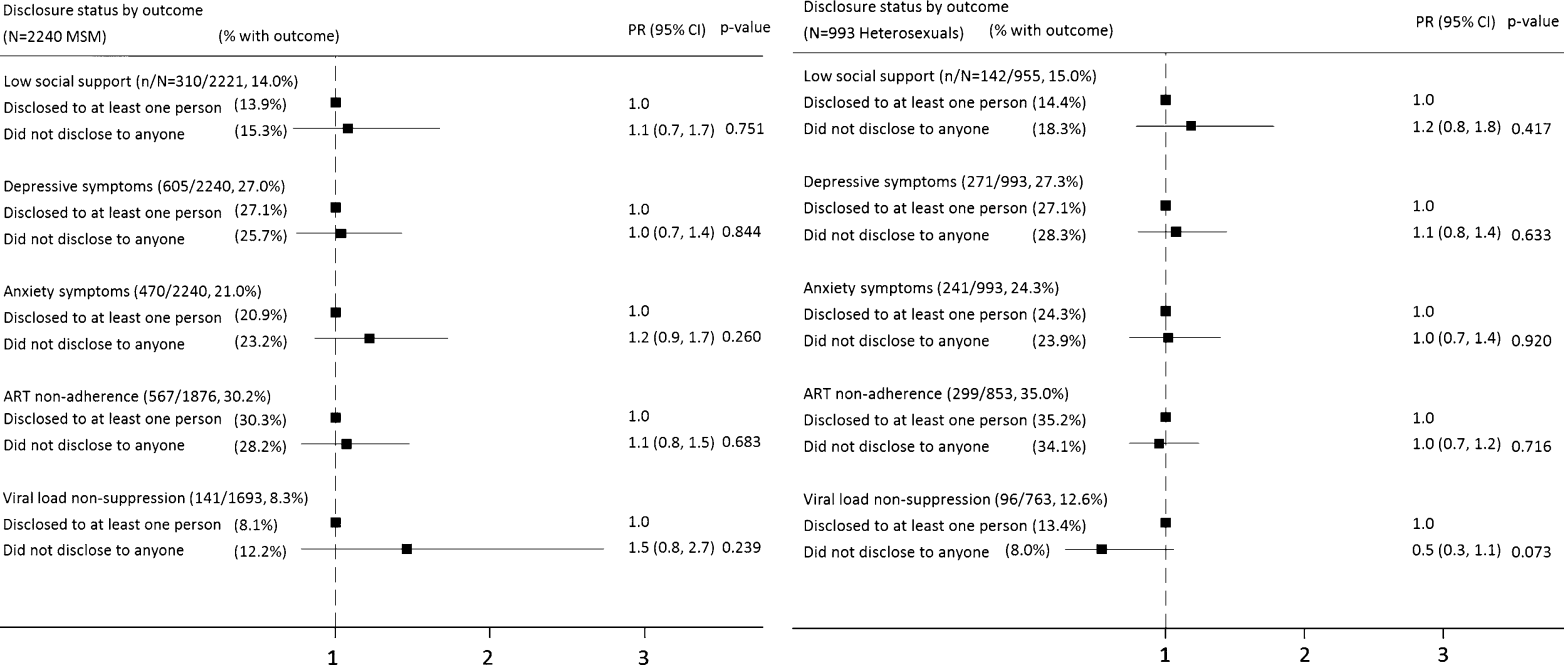
Adjusted associations (prevalence ratios 95 % CIs) between overall non-disclosure and: social support, depressive symptoms, anxiety symptoms, ART non-adherence, and viral load non-suppression on ART, among 2240 MSM (*left panel*) and 993 heterosexual men and women (*right panel*).* PR* prevalence ratio,* CI* confidence interval; multivariable modified Poisson models included gender (for heterosexuals only), age group (<50, ≥50, missing), ethnicity (white, all other), time since HIV diagnosis (<2, 2–10, >10 years), clinic, and ART status (on ART, off ART; for social support, depression, anxiety analyses only); p-values by Wald test; Non-adherence defined as: missed ≥1 ART dose in the past 2 weeks or missed ≥2 consecutive days of ART on more than 1 occasion in the past 3 months; Viral load non-suppression defined as viral load >50 c/mL among those on ART for at least 6 months: n = 1669 MSM, n = 742 heterosexual men and women

Figure 1 shows the prevalence of the dependent variables under study for MSM and heterosexual individuals. There was no significant difference in the prevalence of low social support, depressive or anxiety symptoms between MSM and heterosexuals (p > 0.05 for all, Chi squared test). Compared to MSM, heterosexual men and women had higher prevalence of non-adherence to ART (35.0 % vs. 30.2 %, p < 0.05) and of viral load non-suppression on ART (12.6 vs. 8.3 %, p < 0.05).

Figure 1 also shows adjusted associations between overall non-disclosure versus disclosure to at least one other person among MSM and heterosexuals. Among MSM, overall non-disclosure was not significantly associated with low social support, depression or anxiety symptoms, ART non-adherence, or viral load non-suppression, in unadjusted or adjusted analyses (multivariable models adjusted for age group, ethnicity, time since HIV diagnosis, clinic, and ART status—for social support, depression, anxiety analyses only).

**Fig. 2:**
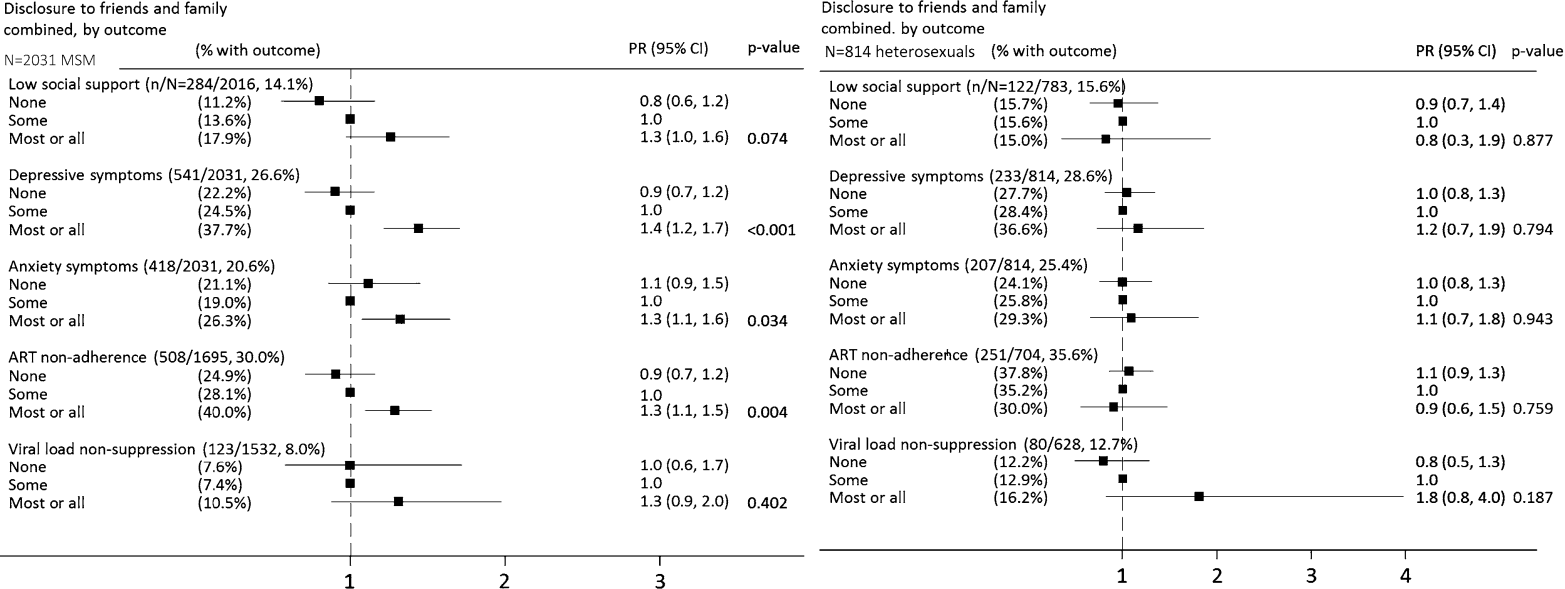
Adjusted associations (prevalence ratios 95 % CIs) between levels of disclosure to friends and family combined and: social support, depressive symptoms, anxiety symptoms, ART non-adherence, and viral load non-suppression on ART, among 2031 MSM (*left panel*) and 814 heterosexual men and women (*right panel*). *PR* prevalence ratio; *CI* confidence interval; multivariable modified Poisson models included gender (for heterosexuals only), age group (<50, ≥50, missing), ethnicity (white, all other), time since HIV diagnosis (<2, 2–10, >10 years), ART status (on ART, off ART; for social support, depression, anxiety analyses only), and clinic; *p* values by Wald test; non-adherence defined as: missed ≥1 ART dose in the past 2 weeks or missed ≥2 consecutive days of ART on more than 1 occasion in the past 3 months; viral load non-suppression defined as viral load >50 c/mL among those on ART for at least 6 months: n = 1489 MSM, n = 605 heterosexuals; combination categories created from disclosure to friends and to family variables as follows; none: none in both, or no answer in one and none in the other; some: some in at least one of the two variables, or most/all in one and either no answer, none, or some in the other; most/all: both variables most/all. Missing for n = 209 MSM, n = 179 heterosexuals. (Prevalence of outcomes in MSM and heterosexuals with missing values in variable disclosure to friends and family was: low social support 12.4 % in MSM, 11.7 % in heterosexual men and women; depression 30.6, 21.2; anxiety 24.9,19.0 %; ART non-adherence 28.2, 26.8 %; viral load non-suppression 11.0, 11.3 %)

There was some evidence to suggest that MSM who had disclosed to ‘most or all’ friends and family were more likely than those who had disclosed to ‘some’ or ‘no’ friends or family to report depression (PR compared to ‘some’ disclosure = 1.4, 95 % CI 1.2–1.7), anxiety (1.3, 1.1–1.6), and ART non-adherence (1.3, 1.1–1.5) (Fig. 2).

For heterosexual men and women, non-disclosure (overall, and level of disclosure to friends and family) was not significantly associated with low social support, depression or anxiety symptoms, ART non-adherence, or viral load non-suppression in unadjusted or adjusted analyses (Figs. 1 and 2).

## Discussion

In this large multicentre cross-sectional study of 3258 people with diagnosed HIV, the prevalence of non-disclosure of HIV status was overall low, but was about three times higher among heterosexual men and women compared to MSM. Non-disclosure was not significantly associated with adverse psychological symptoms, ART non-adherence, or virological non-suppression on ART among MSM or heterosexual individuals.

The prevalence of non-disclosure was higher among heterosexuals of black African or other non-white ethnicity, a pattern corroborated in smaller UK studies [2, 6, 11, 14]. The effect of ethnicity was also apparent among MSM. These ethnic differences, coupled with higher non-disclosure in non UK-born MSM and heterosexuals, may reflect cultural drivers of non-disclosure operating among ethnic minority or migrant populations who may experience perceived or enacted stigma [11, 20]. Attention should be directed to demographic groups with highest non-disclosure so as to better understand circumstances that encourage or discourage it.

The association between age and non-disclosure varies across study context and confidant; in an earlier UK study of 982 white MSM and black African heterosexual men attending HIV clinics, older age was associated with higher non-disclosure to family [11]; two other studies with predominantly black populations (270 clinic attendees in Tanzania, and 269 in southern USA) [8, 10] found the opposite trend, with older participants being more likely to disclose to anyone, including their stable partner. Another US study of 362 young (<24 years) racial minority MSM not in care or newly HIV-diagnosed, found no association between age and non-disclosure of HIV status [7]. In our study, older participants (≥60 years) had higher prevalence of overall non-disclosure. It is possible that older people experience or perceive a greater level of stigma surrounding HIV disclosure than younger people [27]. On the other hand, older people may feel able to better manage HIV without disclosing. Although a higher proportion of older people were diagnosed in the earlier years of the epidemic when prognosis was poor and HIV-related stigma was likely to be greater, this seems unlikely to explain the age association, as an effect of older age was apparent after adjustment for time since HIV diagnosis. In fact, in line with other studies [1, 12, 14, 27], shorter time since diagnosis was independently associated with non-disclosure for MSM and heterosexuals in ASTRA, suggesting that disclosure of HIV status is a gradual process. This finding may highlight the need for health professionals to provide a supportive context soon after diagnosis and to assist in building communication and coping strategies for those who need them. Even though participants not on ART tended to have higher levels of non-disclosure, these associations were explained by shorter time since HIV diagnosis and were not significant in multivariable models. Socio-economic factors such as education and employment status were not associated with non-disclosure among MSM or heterosexuals.

Although gender/sexual orientation differences in the pattern and levels in social disclosure are documented, few studies have investigated these in the context of disclosure to a stable partner/spouse [9]. In ASTRA, non-disclosure to a stable partner was more than twice as high among heterosexuals as among MSM. In line with previous studies [3, 28, 29], our analysis showed that participants who did not know their stable partner’s HIV-serostatus were more likely to have not disclosed their own serostatus, which is likely to reflect the dynamics of mutual disclosure. Longer length of time in the relationship and cohabitation were significantly associated with greater levels of disclosure to a stable partner among heterosexuals; previous studies among black African heterosexual women in the UK and Tanzania found similar results [5, 11], but there is little information on heterosexual men and MSM from the UK. Among MSM in our study, levels of disclosure to a stable partner did not vary according to cohabitation or time in relationship, which may reflect differences in patterns of partnerships and/or sexual relationships among MSM.

The majority of employed participants had not-disclosed to any co-workers, with much higher non-disclosure among heterosexual men and women (84 % in each case). Discrimination against people living with HIV in the workplace is unlawful in the UK [30]. However, the high prevalence of non-disclosure to work colleagues in this sample may be due to prevailing fear of harassment and breach of privacy, or may reflect personal choice regarding disclosure confidants. We highlight the need for employers to enact clear policies, which demonstrate commitment to confidentiality and non-discrimination of HIV-diagnosed employees.

While the therapeutic effect of disclosure as a means of accessing social support has been promoted [9,] evidence on the association between disclosure and measures of mental health status remains mixed. A systematic review of 31 US studies among young (<25 years) people living with HIV found that disclosure was positively associated with higher social support, although younger age may itself be associated with more readily available sources of social support, such as parents, siblings, schoolmates [12]. Similarly, in the international cross-sectional ELLA study (2012–2013) of 1931 HIV-diagnosed women, 11 % had not disclosed to anyone; non-disclosure was significantly associated with lack of social support from family and friends and higher scoring on a scale of severity of barriers to HIV care (BACS). The BACS included measures of lack of psychological support, mental health problems, and stigma [16]. The high prevalence of perceived stigma (78 %) and lack of regular social support (40 %) in ELLA may account for the association between non-disclosure and mental health outcomes, but the relationship may be bidirectional; pre-existing depression and anxiety may predispose individuals to non-disclosure [16]. Conversely, two earlier small cross-sectional studies from the UK (one among 45 black African men and women attending sexual health clinics [2] and one among 95 majority white male HIV outpatient clinic attendees [14]) did not find any association between non-disclosure and social support, but power to examine associations was limited. In our larger study, there was little evidence that individuals who had not disclosed their HIV status were more likely to have low social support or experience psychological symptoms, than those who had disclosed.

Few studies have investigated the association between non-disclosure and ART non-adherence or virological suppression. Our results are similar to those from two previous studies from Canada and the UK showing a lack of an association with non-adherence [20, 31]. In our study, non-disclosure was also not associated with virological non-suppression among MSM or heterosexuals. The lack of association may partly reflect the relative ease of good adherence to current ART without the need to disclose, due to simpler regimens available and lower ART toxicity, or good support from medical teams independent of social disclosure. These findings also appear consistent with the lack of association between non-disclosure and psychological symptoms.

When considering level of disclosure to family and friends among MSM, we found some evidence to suggest that prevalence of depression, anxiety, and ART non-adherence was higher among the relatively small group with high levels of social disclosure compared to those with moderate or no social disclosure. This finding may allude to potential negative consequences of widespread disclosure such as discrimination or rejection from family members and friends. Lower levels of social disclosure could be indicative of a more successful strategy of selecting confidants most likely to provide support and a positive experience of disclosure.

ASTRA is the largest multicentre questionnaire study undertaken among HIV-outpatients in the UK to date, giving reasonable statistical power to examine associations with a factor of low prevalence, such as non-disclosure. However, for our analysis of non-disclosure and viral non-suppression, confidence intervals are wide due to the low prevalence of both factors. Prevalence of non-disclosure may be influenced by non-response; if non-disclosure was more prevalent among those who refused study participation, then our study would underestimate non-disclosure. There was a significant proportion of missing data for the disclosure category sub-questions, but not for the overall non-disclosure question on which our primary analyses are based. In addition, it was not possible from the question wording to ascertain whether disclosure to a stable partner was to the current stable partner, a previous/concurrent partner, or a casual partner, which could underestimate prevalence of non-disclosure to the current partner. To increase the validity of the stable partner non-disclosure measure we restricted the measure to those who reported being in an ongoing relationship only. Due to restricted sample size of those with a stable partner, it was not possible to examine the associations between non-disclosure to a stable partner and measures of social support, mental health, ART adherence, and viral load suppression. We did not include a measure of stigma in the questionnaire, which may have shed further light on associations with non-disclosure. Finally, it should be emphasised that, while epidemiological studies such as ours provide insight into patterns of non-disclosure among a clinic-based population, they are not able to capture the complex circumstances, motivations, and challenges that may surround the issue of disclosure for an HIV-positive individual.

In conclusion, we found that the prevalence of non-disclosure of HIV status to the social circle was overall low, and higher among heterosexuals than MSM. Non-disclosure to anyone in the social network was not associated with higher prevalence of low social support, depression or anxiety symptoms, non-adherence to ART, or viral load non-suppression on ART, suggesting that choosing not to disclose may be a way of coping and is not necessarily linked to adverse psychological consequences or difficulty in managing treatment. These findings are encouraging and may be useful in informing discussions between patients and healthcare professionals about disclosure and ART adherence, and about support available to those who choose not to disclose their status.

## Electronic supplementary material

Below is the link to the electronic supplementary material.

Supplementary material 1 (DOCX 15 kb)
