# Supplementary material for: Non-Disclosure of HIV Status and Associations with Psychological Factors, ART Non-Adherence, and Viral Load Non-Suppression Among People Living with HIV in the UK
Source: AIDS Behav. 2016 Sep 1;21(1):184–95. doi: 10.1007/s10461-016-1541-4 (PMC5216090; doi:10.1007/s10461-016-1541-4)
Supplement: Supplementary file 1 — Supplementary material 1 (DOCX 15 kb) [file 10461_2016_1541_MOESM1_ESM.docx]

**SUPPLEMENTARY DIGITAL CONTENT**

**ebox1: Definition of low social support**

| The modified the Duke UNC FSSQ uses the following statements   1. “I have people who care what happens to me” 2. “I get love and affection” 3. “I get chances to talk to someone I trust about my personal problems” 4. “I get invitations to go out and do things with people” 5. “I get help when I am sick in bed”   The above statements are scored on a Likert scale (from 1 to 5):  As much as I would like = 5  Almost as much as I would like = 4  Some, but would like more = 3  Less than I would like = 2  Much less than I would like = 1  Statement scores for each participant were summed (range 5-25). To replace missing statement responses when at least one statement response was available, we used the average score of statements completed for that particular participant. A total score of 12 or less was considered ‘low social support’. |
| --- |
